# Di-μ-sulfato-bis­[diaqua­(1*H*-imidazo[4,5-*f*][1,10]phenanthroline)iron(II)] dihydrate

**DOI:** 10.1107/S1600536810032496

**Published:** 2010-08-18

**Authors:** Ming-Xing Yang, Shen Lin, Hui-Ying Shen, Li-Juan Chen

**Affiliations:** a College of Chemistry and Materials Science, Fujian Normal University, Fuzhou 350007, People’s Republic of China; b State Key Laboratory of Structural Chemistry, Fujian Institute of Research on the Structure of Matter, Chinese Academy of Science, Fuzhou 350002, People’s Republic of China

## Abstract

The title dinuclear Fe^II^ complex, [Fe_2_(SO_4_)_2_(C_13_H_8_N_4_)_2_(H_2_O)_4_]·2H_2_O, is centrosymmetric. Two sulfate anions bridge two Fe^II^ cations to form the binuclear complex. Each Fe^II^ cation is coordinated by two N atoms from a 1*H*-imidazo[4,5-*f*][1,10]phenanthroline (IP) ligand, two O atoms from two sulfate anions and two water mol­ecules in a distorted octa­hedral geometry. Extensive O—H⋯O, N—H⋯O and O—H⋯N hydrogen bonding is present in the crystal structure. Weak π–π stacking is observed between parallel IP ring systems, the face-to-face separation being 3.428 (14) Å.

## Related literature

For metal complexes with the 1*H*-imidazo[4,5-*f*][1,10]phenanthroline (IP) ligand, see: Liu *et al.* (2009 ▶); Stephenson *et al.* (2008 ▶); Wu *et al.* (1997 ▶); Yang *et al.* (2010 ▶); Yu (2009 ▶). For the synthesis of IP, see: Wu *et al.* (1997 ▶).
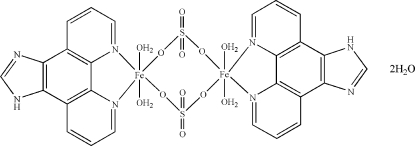


## Experimental

### 

#### Crystal data

[Fe_2_(SO_4_)_2_(C_13_H_8_N_4_)_2_(H_2_O)_4_]·2H_2_O
*M* *_r_* = 852.38
Monoclinic, 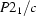

*a* = 10.2879 (9) Å
*b* = 9.0738 (8) Å
*c* = 17.0089 (16) Å
β = 98.892 (5)°
*V* = 1568.7 (2) Å^3^
*Z* = 2
Mo *K*α radiation
μ = 1.14 mm^−1^
*T* = 293 K
0.20 × 0.20 × 0.10 mm

#### Data collection

Rigaku Mercury CCD diffractometer
Absorption correction: multi-scan (*CrystalClear*; Rigaku, 2002 ▶) *T*_min_ = 0.673, *T*_max_ = 1.000
11834 measured reflections
3500 independent reflections
2884 reflections with *I* > 2σ(*I*)
*R*_int_ = 0.040

#### Refinement

*R*[*F* ^2^ > 2σ(*F* ^2^)] = 0.040
*wR*(*F* ^2^) = 0.099
*S* = 1.05
3500 reflections
259 parameters
9 restraints
H atoms treated by a mixture of independent and constrained refinement
Δρ_max_ = 0.44 e Å^−3^
Δρ_min_ = −0.47 e Å^−3^

### 

Data collection: *CrystalClear* (Rigaku, 2002 ▶); cell refinement: *CrystalClear*; data reduction: *CrystalClear*; program(s) used to solve structure: *SHELXS97* (Sheldrick, 2008 ▶); program(s) used to refine structure: *SHELXL97* (Sheldrick, 2008 ▶; molecular graphics: *SHELXTL* (Sheldrick, 2008 ▶); software used to prepare material for publication: *SHELXTL*.

## Supplementary Material

Crystal structure: contains datablocks global, I. DOI: 10.1107/S1600536810032496/xu5012sup1.cif

Structure factors: contains datablocks I. DOI: 10.1107/S1600536810032496/xu5012Isup2.hkl

Additional supplementary materials:  crystallographic information; 3D view; checkCIF report

## Figures and Tables

**Table 1:** Selected bond lengths (Å)

Fe1—N1	2.175 (2)
Fe1—N2	2.172 (2)
Fe1—O1	2.0865 (17)
Fe1—O2^i^	2.1065 (18)
Fe1—O5	2.197 (2)
Fe1—O6	2.108 (2)

Symmetry code: (i) 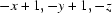.

**Table 2:** Hydrogen-bond geometry (Å, °)

*D*—H⋯*A*	*D*—H	H⋯*A*	*D*⋯*A*	*D*—H⋯*A*
N4—H4*B*⋯O4^ii^	0.86	2.05	2.891 (3)	164
O5—H1⋯N3^iii^	0.86 (4)	2.00 (4)	2.807 (3)	157 (4)
O5—H2⋯O3	0.84 (2)	1.97 (2)	2.773 (3)	159 (2)
O6—H3⋯O3^i^	0.84 (3)	1.93 (2)	2.706 (3)	152 (3)
O6—H4⋯O7	0.84 (2)	1.79 (2)	2.633 (3)	178 (4)
O7—H5⋯O4^iv^	0.84 (2)	1.99 (2)	2.803 (3)	163 (3)
O7—H6⋯O3^v^	0.84 (2)	1.99 (2)	2.823 (3)	169 (3)

Symmetry codes: (i) 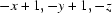; (ii) 
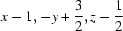
; (iii) 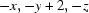; (iv) 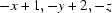; (v) 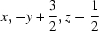.

**Fig. 1.**
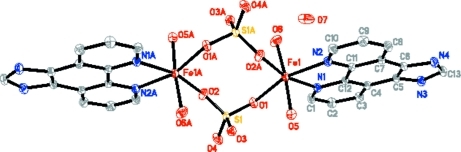
The molecular structure of the title complound, showing 30% probability displacement ellipsoids with atoms numbering. H atoms have been omitted for clarity.

**Fig. 2.**
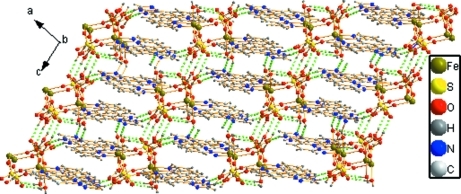
The three-dimensional hydrogen bonding network along the b axis.

## supplementary crystallographic information

### Comment

Transitional metal complexes of 1,10-Phenanthroline's derivatives still continue to attract intense interest not only because of their fascinating architectures but also because of the intriguing properties, such as magnetic, biological activity and optical properties. The IP ligand,as one of 1,10-Phenanthroline's derivatives, has recently gained a lot of interest with respect to synthesis of its novel metal compounds. It has been used to construct coordination frameworks by the direct interaction with metal ions or as secondary ligands to form discrete polynuclear, one-dimensional, two-dimensional and three-dimensional coordination networks. Its metal complexes are focused on Ru, Co, Ni, Cd, Cu, Mn and Zn complexes (Liu *et al.*, 2009; Stephenson *et al.*, 2008; Wu *et al.*, 1997; Yang *et al.*, 2010; Yu, 2009;). As an extension of the work on the structural characterization of IP complexes, the preparation and crystal structure of the title Fe^II^ complex is reported here.

In centrosymmetric dinuclear complex, the sulfate acts as an O—S—O bridge across two Fe^II^ cation, determining the formation of a dimer (Fig. 1).The Fe^II^ cation has a distorted octahedral coordination completed by two nitrogen atoms from one IP ligand, two oxygen atoms from water and two oxygen atoms from two sulfuric anions. The equatorial plane of the octahedron is defined by N1, O6, O2, O5 around Fe1, and the axial coordination sites are occupied by N2 and O1 atoms.

Strong hydrogen bonds exist in the structure (Table 2). The complicated three-dimensional hydrogen bonding network is shown in Fig. 2. The uncoordinated water molecular is a hydrogen bond acceptor from the coordinated water and a hydrogen bond donor to two O atoms of two sulfuric anions in two neighboring [Fe_2_(SO_4_)_2_(IP)_2_(H_2_O)_2_] species. The [Fe_2_(SO_4_)_2_(IP)_2_(H_2_O)_2_] molecules also form hydrogen bonds between themselves through O—H···N and N—H···O interactions from the imidazolyl ring. So [Fe_2_(SO_4_)_2_(C_13_H_8_N_4_)_2_(H_2_O)_2_] molecules and the uncoordinated water are connected by O—H···O, O—H···N and N—H···O hydrogen bonds into a three-dimensional network structure. There is also a π-π stacking interaction between the IP ligands of the neighboring [Fe_2_(SO_4_)_2_(IP)_2_(H_2_O)_2_] species with an interplanar separation of about 3.428 (14) Å [symmetry code = -*x*, 2 - *y*, -*z*].

### Experimental

The IP was synthesized according to reference of Wu *et al.* (1997). A mixture of FeSO_4_.7H_2_O, benzene-1,4-dicarboxylic acid, IP and H_2_O in a molar ratio 1:1:1:556 was stirred for 1 h, then sealed in an 18 ml Teflon-lined stainless steel reactor and heated for 3 d at 433 K and autogeneous pressure. After allowing the reaction mixture to cool down to room temperature, yellow crystals were obtained.

### Refinement

Water H atoms were located in a difference Fourier map and refined isotropically with restrained O—H distance = 0.84 (1) Å and H···H distance = 1.44 (1) Å. The other H atoms were generated geometrically with C—H = 0.93 and N—H = 0.86 Å, *U*_iso_(H) = 1.2*U*_eq_(C,*N*).

### Crystal data

**Table:**

[Fe_2_(SO_4_)_2_(C_13_H_8_N_4_)_2_(H_2_O)_4_]·2H_2_O	*F*(000) = 872
*M**_r_* = 852.38	*D*_x_ = 1.805 Mg m^−^^3^
Monoclinic, *P*2_1_/*c*	Mo *K*α radiation, λ = 0.71073 Å
Hall symbol: -P 2ybc	Cell parameters from 3717 reflections
*a* = 10.2879 (9) Å	θ = 3.0–27.5°
*b* = 9.0738 (8) Å	µ = 1.14 mm^−^^1^
*c* = 17.0089 (16) Å	*T* = 293 K
β = 98.892 (5)°	Prism, yellow
*V* = 1568.7 (2) Å^3^	0.20 × 0.20 × 0.10 mm
*Z* = 2	

### Data collection

**Table:**

Rigaku Mercury CCD diffractometer	3500 independent reflections
Radiation source: fine-focus sealed tube	2884 reflections with *I* > 2σ(*I*)
graphite	*R*_int_ = 0.040
Detector resolution: 14.6306 pixels mm^-1^	θ_max_ = 27.5°, θ_min_ = 2.6°
ω scan	*h* = −13→13
Absorption correction: multi-scan (*CrystalClear*; Rigaku, 2002)	*k* = −11→11
*T*_min_ = 0.673, *T*_max_ = 1.000	*l* = −22→21
11834 measured reflections	

### Refinement

**Table:**

Refinement on *F*^2^	Primary atom site location: structure-invariant direct methods
Least-squares matrix: full	Secondary atom site location: difference Fourier map
*R*[*F*^2^ > 2σ(*F*^2^)] = 0.040	Hydrogen site location: inferred from neighbouring sites
*wR*(*F*^2^) = 0.099	H atoms treated by a mixture of independent and constrained refinement
*S* = 1.05	*w* = 1/[σ^2^(*F*_o_^2^) + (0.0483*P*)^2^ + 0.6512*P*] where *P* = (*F*_o_^2^ + 2*F*_c_^2^)/3
3500 reflections	(Δ/σ)_max_ = 0.001
259 parameters	Δρ_max_ = 0.44 e Å^−^^3^
9 restraints	Δρ_min_ = −0.47 e Å^−^^3^

### Special details

**Table:**

Geometry. All e.s.d.'s (except the e.s.d. in the dihedral angle between two l.s. planes) are estimated using the full covariance matrix. The cell e.s.d.'s are taken into account individually in the estimation of e.s.d.'s in distances, angles and torsion angles; correlations between e.s.d.'s in cell parameters are only used when they are defined by crystal symmetry. An approximate (isotropic) treatment of cell e.s.d.'s is used for estimating e.s.d.'s involving l.s. planes.
Refinement. Refinement of *F*^2^ against ALL reflections. The weighted *R*-factor *wR* and goodness of fit *S* are based on *F*^2^, conventional *R*-factors *R* are based on *F*, with *F* set to zero for negative *F*^2^. The threshold expression of *F*^2^ > σ(*F*^2^) is used only for calculating *R*-factors(gt) *etc*. and is not relevant to the choice of reflections for refinement. *R*-factors based on *F*^2^ are statistically about twice as large as those based on *F*, and *R*- factors based on ALL data will be even larger.

### Fractional atomic coordinates and isotropic or equivalent isotropic displacement parameters (Å^2^)

**Table:**

	*x*	*y*	*z*	*U*_iso_*/*U*_eq_	
Fe1	0.31610 (3)	0.68065 (4)	−0.03898 (2)	0.02489 (13)	
S1	0.53353 (6)	0.60216 (6)	0.11438 (4)	0.02472 (16)	
O1	0.46200 (17)	0.71051 (18)	0.05947 (11)	0.0298 (4)	
O2	0.63573 (18)	0.53106 (19)	0.07634 (12)	0.0348 (4)	
O3	0.44027 (17)	0.48994 (19)	0.13588 (11)	0.0314 (4)	
O4	0.5938 (2)	0.6791 (2)	0.18664 (12)	0.0402 (5)	
O5	0.20448 (19)	0.5633 (2)	0.04180 (11)	0.0347 (4)	
O6	0.4329 (2)	0.7669 (2)	−0.11949 (14)	0.0465 (5)	
O7	0.4090 (4)	1.0134 (3)	−0.20216 (16)	0.0697 (8)	
N1	0.2437 (2)	0.9048 (2)	−0.03242 (12)	0.0256 (5)	
N2	0.1277 (2)	0.6775 (2)	−0.11677 (12)	0.0261 (5)	
N3	−0.1442 (2)	1.2057 (2)	−0.13981 (14)	0.0339 (5)	
N4	−0.2454 (2)	1.0126 (2)	−0.20243 (13)	0.0329 (5)	
H4B	−0.3055	0.9614	−0.2308	0.040*	
C1	0.3070 (2)	1.0173 (3)	0.00618 (16)	0.0297 (6)	
H1A	0.3891	1.0002	0.0362	0.036*	
C2	0.2562 (3)	1.1597 (3)	0.00372 (17)	0.0333 (6)	
H2B	0.3047	1.2358	0.0307	0.040*	
C3	0.1336 (3)	1.1870 (3)	−0.03888 (16)	0.0301 (6)	
H3C	0.0971	1.2809	−0.0399	0.036*	
C4	0.0648 (2)	1.0710 (3)	−0.08061 (15)	0.0252 (5)	
C5	−0.0639 (2)	1.0827 (3)	−0.12661 (15)	0.0265 (5)	
C6	−0.1251 (2)	0.9628 (3)	−0.16564 (14)	0.0264 (5)	
C7	−0.0665 (2)	0.8197 (3)	−0.16491 (14)	0.0244 (5)	
C8	−0.1246 (3)	0.6951 (3)	−0.20433 (16)	0.0324 (6)	
H8A	−0.2079	0.7007	−0.2345	0.039*	
C9	−0.0566 (3)	0.5653 (3)	−0.19772 (17)	0.0339 (6)	
H9A	−0.0940	0.4804	−0.2222	0.041*	
C10	0.0698 (3)	0.5616 (3)	−0.15377 (16)	0.0307 (6)	
H10A	0.1156	0.4728	−0.1504	0.037*	
C11	0.0604 (2)	0.8071 (3)	−0.12085 (14)	0.0235 (5)	
C12	0.1247 (2)	0.9309 (2)	−0.07685 (14)	0.0225 (5)	
C13	−0.2508 (3)	1.1563 (3)	−0.18553 (17)	0.0373 (7)	
H13A	−0.3226	1.2156	−0.2042	0.045*	
H2	0.266 (2)	0.521 (3)	0.0720 (16)	0.061 (11)*	
H3	0.484 (3)	0.705 (2)	−0.1358 (18)	0.045 (9)*	
H1	0.165 (4)	0.631 (4)	0.064 (2)	0.113 (19)*	
H5	0.410 (4)	1.1018 (18)	−0.187 (2)	0.087 (15)*	
H4	0.425 (4)	0.847 (2)	−0.145 (2)	0.085 (14)*	
H6	0.413 (4)	1.001 (4)	−0.2509 (9)	0.080 (14)*	

### Atomic displacement parameters (Å^2^)

**Table:**

	*U*^11^	*U*^22^	*U*^33^	*U*^12^	*U*^13^	*U*^23^
Fe1	0.0230 (2)	0.02412 (19)	0.0261 (2)	0.00472 (14)	−0.00072 (15)	0.00128 (14)
S1	0.0243 (3)	0.0228 (3)	0.0247 (3)	0.0051 (2)	−0.0034 (2)	−0.0018 (2)
O1	0.0280 (9)	0.0261 (8)	0.0323 (10)	0.0032 (7)	−0.0052 (8)	0.0037 (7)
O2	0.0306 (10)	0.0254 (8)	0.0496 (12)	0.0044 (8)	0.0099 (9)	−0.0017 (9)
O3	0.0305 (9)	0.0295 (9)	0.0342 (10)	0.0033 (8)	0.0046 (8)	0.0048 (8)
O4	0.0499 (12)	0.0330 (10)	0.0315 (10)	0.0058 (9)	−0.0135 (9)	−0.0075 (8)
O5	0.0322 (10)	0.0370 (10)	0.0345 (11)	0.0040 (9)	0.0038 (9)	0.0017 (9)
O6	0.0586 (14)	0.0318 (10)	0.0556 (14)	0.0146 (10)	0.0291 (12)	0.0116 (10)
O7	0.137 (3)	0.0325 (12)	0.0432 (15)	0.0165 (15)	0.0272 (17)	0.0078 (11)
N1	0.0226 (10)	0.0277 (10)	0.0254 (11)	0.0019 (8)	0.0008 (9)	−0.0009 (9)
N2	0.0263 (10)	0.0248 (10)	0.0258 (11)	0.0028 (8)	0.0000 (9)	−0.0003 (9)
N3	0.0335 (12)	0.0325 (11)	0.0342 (13)	0.0094 (10)	0.0004 (10)	0.0014 (10)
N4	0.0255 (11)	0.0399 (12)	0.0304 (12)	0.0024 (10)	−0.0050 (9)	−0.0004 (10)
C1	0.0215 (12)	0.0322 (13)	0.0331 (14)	−0.0005 (10)	−0.0023 (11)	−0.0029 (11)
C2	0.0327 (14)	0.0277 (12)	0.0380 (15)	−0.0059 (11)	0.0006 (12)	−0.0045 (12)
C3	0.0341 (14)	0.0225 (11)	0.0332 (14)	0.0016 (10)	0.0037 (12)	−0.0001 (10)
C4	0.0258 (12)	0.0255 (11)	0.0241 (12)	0.0031 (10)	0.0035 (10)	0.0017 (10)
C5	0.0278 (12)	0.0257 (12)	0.0254 (13)	0.0066 (10)	0.0020 (10)	0.0020 (10)
C6	0.0227 (12)	0.0337 (13)	0.0220 (12)	0.0049 (10)	0.0013 (10)	0.0013 (10)
C7	0.0231 (12)	0.0268 (12)	0.0230 (12)	0.0024 (10)	0.0027 (10)	0.0000 (10)
C8	0.0245 (12)	0.0376 (14)	0.0330 (15)	−0.0006 (11)	−0.0020 (11)	−0.0037 (11)
C9	0.0359 (14)	0.0303 (13)	0.0349 (15)	−0.0052 (12)	0.0034 (12)	−0.0071 (11)
C10	0.0336 (14)	0.0247 (11)	0.0333 (14)	0.0024 (11)	0.0037 (12)	−0.0009 (11)
C11	0.0227 (11)	0.0260 (11)	0.0219 (12)	0.0025 (10)	0.0036 (10)	0.0003 (9)
C12	0.0219 (11)	0.0229 (11)	0.0225 (12)	0.0031 (9)	0.0027 (10)	−0.0004 (9)
C13	0.0344 (15)	0.0424 (15)	0.0325 (15)	0.0172 (12)	−0.0027 (12)	0.0040 (12)

### Geometric parameters (Å, °)

**Table:**

Fe1—N1	2.175 (2)	N4—C13	1.338 (4)
Fe1—N2	2.172 (2)	N4—C6	1.373 (3)
Fe1—O1	2.0865 (17)	N4—H4B	0.8600
Fe1—O2^i^	2.1065 (18)	C1—C2	1.392 (4)
Fe1—O5	2.197 (2)	C1—H1A	0.9300
Fe1—O6	2.108 (2)	C2—C3	1.377 (4)
S1—O4	1.4649 (18)	C2—H2B	0.9300
S1—O2	1.4665 (19)	C3—C4	1.399 (3)
S1—O1	1.4723 (17)	C3—H3C	0.9300
S1—O3	1.4831 (19)	C4—C12	1.411 (3)
O5—H2	0.84 (2)	C4—C5	1.433 (3)
O5—H1	0.86 (4)	C5—C6	1.375 (3)
O6—H3	0.84 (3)	C6—C7	1.431 (3)
O6—H4	0.84 (2)	C7—C8	1.400 (3)
O7—H5	0.842 (19)	C7—C11	1.405 (3)
O7—H6	0.844 (18)	C8—C9	1.366 (4)
N1—C1	1.329 (3)	C8—H8A	0.9300
N1—C12	1.356 (3)	C9—C10	1.397 (4)
N2—C10	1.320 (3)	C9—H9A	0.9300
N2—C11	1.361 (3)	C10—H10A	0.9300
N3—C13	1.320 (4)	C11—C12	1.452 (3)
N3—C5	1.387 (3)	C13—H13A	0.9300
			
O1—Fe1—O2^i^	100.77 (7)	N1—C1—C2	123.0 (2)
O1—Fe1—O6	93.52 (9)	N1—C1—H1A	118.5
O2^i^—Fe1—O6	87.60 (8)	C2—C1—H1A	118.5
O1—Fe1—N2	162.62 (8)	C3—C2—C1	119.5 (2)
O2^i^—Fe1—N2	91.93 (7)	C3—C2—H2B	120.3
O6—Fe1—N2	98.86 (9)	C1—C2—H2B	120.3
O1—Fe1—N1	92.70 (7)	C2—C3—C4	118.8 (2)
O2^i^—Fe1—N1	165.19 (8)	C2—C3—H3C	120.6
O6—Fe1—N1	85.43 (8)	C4—C3—H3C	120.6
N2—Fe1—N1	76.29 (7)	C3—C4—C12	118.1 (2)
O1—Fe1—O5	86.66 (7)	C3—C4—C5	125.0 (2)
O2^i^—Fe1—O5	85.22 (8)	C12—C4—C5	116.9 (2)
O6—Fe1—O5	172.72 (8)	C6—C5—N3	110.0 (2)
N2—Fe1—O5	82.59 (8)	C6—C5—C4	121.4 (2)
N1—Fe1—O5	101.83 (8)	N3—C5—C4	128.7 (2)
O4—S1—O2	109.92 (12)	N4—C6—C5	105.8 (2)
O4—S1—O1	108.61 (10)	N4—C6—C7	130.6 (2)
O2—S1—O1	109.63 (11)	C5—C6—C7	123.5 (2)
O4—S1—O3	109.03 (12)	C8—C7—C11	118.8 (2)
O2—S1—O3	110.00 (11)	C8—C7—C6	125.5 (2)
O1—S1—O3	109.62 (10)	C11—C7—C6	115.7 (2)
S1—O1—Fe1	130.50 (11)	C9—C8—C7	118.8 (2)
S1—O2—Fe1^i^	138.81 (12)	C9—C8—H8A	120.6
Fe1—O5—H2	100 (2)	C7—C8—H8A	120.6
Fe1—O5—H1	105 (3)	C8—C9—C10	119.2 (2)
H2—O5—H1	114.9 (18)	C8—C9—H9A	120.4
Fe1—O6—H3	114.5 (19)	C10—C9—H9A	120.4
Fe1—O6—H4	129 (2)	N2—C10—C9	123.4 (2)
H3—O6—H4	114.8 (17)	N2—C10—H10A	118.3
H5—O7—H6	115.9 (18)	C9—C10—H10A	118.3
C1—N1—C12	118.2 (2)	N2—C11—C7	121.4 (2)
C1—N1—Fe1	126.73 (16)	N2—C11—C12	117.0 (2)
C12—N1—Fe1	114.93 (15)	C7—C11—C12	121.6 (2)
C10—N2—C11	118.4 (2)	N1—C12—C4	122.2 (2)
C10—N2—Fe1	126.54 (16)	N1—C12—C11	116.9 (2)
C11—N2—Fe1	114.76 (15)	C4—C12—C11	120.8 (2)
C13—N3—C5	104.0 (2)	N3—C13—N4	113.5 (2)
C13—N4—C6	106.7 (2)	N3—C13—H13A	123.2
C13—N4—H4B	126.7	N4—C13—H13A	123.2
C6—N4—H4B	126.7		
			
O4—S1—O1—Fe1	−162.26 (14)	C3—C4—C5—N3	0.5 (4)
O2—S1—O1—Fe1	77.62 (16)	C12—C4—C5—N3	179.7 (3)
O3—S1—O1—Fe1	−43.22 (18)	C13—N4—C6—C5	0.8 (3)
O2^i^—Fe1—O1—S1	−28.46 (16)	C13—N4—C6—C7	−178.7 (3)
O6—Fe1—O1—S1	−116.68 (15)	N3—C5—C6—N4	−0.8 (3)
N2—Fe1—O1—S1	107.8 (2)	C4—C5—C6—N4	179.4 (2)
N1—Fe1—O1—S1	157.73 (15)	N3—C5—C6—C7	178.8 (2)
O5—Fe1—O1—S1	56.03 (15)	C4—C5—C6—C7	−1.0 (4)
O4—S1—O2—Fe1^i^	123.58 (18)	N4—C6—C7—C8	0.1 (5)
O1—S1—O2—Fe1^i^	−117.10 (18)	C5—C6—C7—C8	−179.4 (3)
O3—S1—O2—Fe1^i^	3.5 (2)	N4—C6—C7—C11	179.6 (3)
O1—Fe1—N1—C1	18.0 (2)	C5—C6—C7—C11	0.2 (4)
O2^i^—Fe1—N1—C1	−137.6 (3)	C11—C7—C8—C9	0.8 (4)
O6—Fe1—N1—C1	−75.3 (2)	C6—C7—C8—C9	−179.6 (3)
N2—Fe1—N1—C1	−175.6 (2)	C7—C8—C9—C10	−1.7 (4)
O5—Fe1—N1—C1	105.1 (2)	C11—N2—C10—C9	0.7 (4)
O1—Fe1—N1—C12	−166.31 (17)	Fe1—N2—C10—C9	174.5 (2)
O2^i^—Fe1—N1—C12	38.2 (4)	C8—C9—C10—N2	1.0 (4)
O6—Fe1—N1—C12	100.37 (18)	C10—N2—C11—C7	−1.6 (4)
N2—Fe1—N1—C12	0.08 (17)	Fe1—N2—C11—C7	−176.12 (19)
O5—Fe1—N1—C12	−79.15 (18)	C10—N2—C11—C12	177.6 (2)
O1—Fe1—N2—C10	−123.8 (3)	Fe1—N2—C11—C12	3.1 (3)
O2^i^—Fe1—N2—C10	13.4 (2)	C8—C7—C11—N2	0.9 (4)
O6—Fe1—N2—C10	101.3 (2)	C6—C7—C11—N2	−178.7 (2)
N1—Fe1—N2—C10	−175.7 (2)	C8—C7—C11—C12	−178.3 (2)
O5—Fe1—N2—C10	−71.5 (2)	C6—C7—C11—C12	2.1 (4)
O1—Fe1—N2—C11	50.2 (3)	C1—N1—C12—C4	−2.6 (4)
O2^i^—Fe1—N2—C11	−172.64 (18)	Fe1—N1—C12—C4	−178.72 (19)
O6—Fe1—N2—C11	−84.77 (19)	C1—N1—C12—C11	177.6 (2)
N1—Fe1—N2—C11	−1.72 (17)	Fe1—N1—C12—C11	1.5 (3)
O5—Fe1—N2—C11	102.43 (18)	C3—C4—C12—N1	2.2 (4)
C12—N1—C1—C2	0.8 (4)	C5—C4—C12—N1	−177.0 (2)
Fe1—N1—C1—C2	176.3 (2)	C3—C4—C12—C11	−178.0 (2)
N1—C1—C2—C3	1.5 (4)	C5—C4—C12—C11	2.7 (4)
C1—C2—C3—C4	−1.8 (4)	N2—C11—C12—N1	−3.1 (3)
C2—C3—C4—C12	0.1 (4)	C7—C11—C12—N1	176.1 (2)
C2—C3—C4—C5	179.3 (3)	N2—C11—C12—C4	177.1 (2)
C13—N3—C5—C6	0.4 (3)	C7—C11—C12—C4	−3.7 (4)
C13—N3—C5—C4	−179.8 (3)	C5—N3—C13—N4	0.1 (3)
C3—C4—C5—C6	−179.7 (3)	C6—N4—C13—N3	−0.6 (3)
C12—C4—C5—C6	−0.5 (4)		

Symmetry codes: (i) −*x*+1, −*y*+1, −*z*.

### Hydrogen-bond geometry (Å, °)

**Table:**

*D*—H···*A*	*D*—H	H···*A*	*D*···*A*	*D*—H···*A*
N4—H4B···O4^ii^	0.86	2.05	2.891 (3)	164
O5—H1···N3^iii^	0.86 (4)	2.00 (4)	2.807 (3)	157 (4)
O5—H2···O3	0.84 (2)	1.97 (2)	2.773 (3)	159 (2)
O6—H3···O3^i^	0.84 (3)	1.93 (2)	2.706 (3)	152 (3)
O6—H4···O7	0.84 (2)	1.79 (2)	2.633 (3)	178 (4)
O7—H5···O4^iv^	0.84 (2)	1.99 (2)	2.803 (3)	163 (3)
O7—H6···O3^v^	0.84 (2)	1.99 (2)	2.823 (3)	169 (3)

Symmetry codes: (ii) *x*−1, −*y*+3/2, *z*−1/2; (iii) −*x*, −*y*+2, −*z*; (i) −*x*+1, −*y*+1, −*z*; (iv) −*x*+1, −*y*+2, −*z*; (v) *x*, −*y*+3/2, *z*−1/2.
